# Heterogeneity of macrophage activation syndrome and treatment progression

**DOI:** 10.3389/fimmu.2024.1389710

**Published:** 2024-04-26

**Authors:** Yuanji Dong, Ting Wang, Huaxiang Wu

**Affiliations:** ^1^ Department of Rheumatology, The Second Affiliated Hospital of Zhejiang University School of Medicine, Hangzhou, Zhejiang, China; ^2^ Department of Respiratory Disease, Thoracic Disease Center, The First Affiliated Hospital, College of Medicine, Zhejiang University, Hangzhou, Zhejiang, China

**Keywords:** macrophage activation syndrome, autoimmune inflammatory rheumatic diseases, cytokine storms, heterogeneity, treatment progression

## Abstract

Macrophage activation syndrome (MAS) is a rare complication of autoimmune inflammatory rheumatic diseases (AIIRD) characterized by a progressive and life-threatening condition with features including cytokine storm and hemophagocytosis. Predisposing factors are typically associated with microbial infections, genetic factors (distinct from typical genetically related hemophagocytic lymphohistiocytosis (HLH)), and inappropriate immune system overactivation. Clinical features include unremitting fever, generalized rash, hepatosplenomegaly, lymphadenopathy, anemia, worsening liver function, and neurological involvement. MAS can occur in various AIIRDs, including but not limited to systemic juvenile idiopathic arthritis (sJIA), adult-onset Still’s disease (AOSD), systemic lupus erythematosus (SLE), Kawasaki disease (KD), juvenile dermatomyositis (JDM), rheumatoid arthritis (RA), and Sjögren’s syndrome (SS), etc. Although progress has been made in understanding the pathogenesis and treatment of MAS, it is important to recognize the differences between different diseases and the various treatment options available. This article summarizes the cell types and cytokines involved in MAS-related diseases, the heterogeneity, and treatment options, while also comparing it to genetically related HLH.

## 1 Introduction

Macrophage activation syndrome (MAS) is the term used to describe hemophagocytic lymphohistiocytosis (HLH) in the context of rheumatological disorders. It is one of the causes of HLH syndrome (1). MAS was initially identified in pediatric patients, but it has now been widely reported to occur also in adults (2). MAS is believed to be characterized by inappropriate and sustained activation of cytotoxic cells (such as CD8+ T cells and natural killer cells) as well as myeloid cells (macrophages, dendritic cells), leading to a systemic cytokine storm including interferon-γ (IFN-γ), Interleukin-18 (IL-18), Tumor necrosis factor-α (TNF-α), IL-6, IL-1, etc. (3–6). The exact etiology of MAS is not completely understood, but it is often associated with certain triggering factors, including microbial infections (e.g. EBV, CMV), genetic factors (30-40% of MAS/secondary HLH (sHLH) patients have heterozygous defects in perforin-pathway familial HLH genes), and medications (e.g. CAR-T), but also that no associated triggers may be identified (7, 8). The clinical manifestations of MAS commonly include high fever, rash, thrombocytopenia, anemia, and abnormal liver function. The condition can rapidly progress and lead to multiple organ dysfunction, resulting in a high mortality rate (2, 4). However, compared to the overall mortality rate of 41% in adult sHLH and the high mortality rate ranging from 42% to 88% in tumor-associated HLH, autoimmune inflammatory rheumatic diseases (AIIRD)-MAS generally has a lower overall mortality rate, fluctuating between 5% and 39% (2). The mortality rate for systemic juvenile idiopathic arthritis (sJIA)-MAS is approximately 8-23% (9), adult-onset Still’s disease (AOSD)-MAS is around 10%-22% (10), systemic lupus erythematosus (SLE)-MAS is approximately 5-35% (9), Kawasaki disease (KD)-MAS is around 13% (11), and juvenile dermatomyositis (JDM)-MAS is approximately 16.7% (12). Mortality rates for other AIIRD are limited. Another study suggests that the 90-day all-cause mortality rate in patients with rheumatic disease-associated MAS is 22.9% (13). Currently, there is a lack of specific diagnostic criteria for AIIRD-MAS, and clinicians often rely on diagnostic criteria for HLH or sJIA-MAS, including the HScore and MScore. The former (HScore), which applies to acquired HLH in adults, has a diagnostic cutoff value of greater than 169 (with a sensitivity of 93% and specificity of 86%). The latter (MScore), which applies to sJIA-MAS, has a diagnostic cutoff value of less than -2.1 (with a sensitivity of 85% and specificity of 95%) (14–18). In addition, researchers have developed a simple indicator to identify sJIA-MAS, a ferritin/erythrocyte sedimentation rate (ESR) ratio greater than 21.5 (with a sensitivity of 82% and specificity of 78%) (19). Both MScore and Ferritin/ESR ratios are used to distinguish active sJIA from sJIA-MAS. Recently, the European League Against Rheumatism/American College of Rheumatology (EULAR/ACR) recommended points to consider at the early stages of diagnosis and management of suspected HLH/MAS, which is very instructive for AIIRD-MAS (4).

For the treatment of AIIRD-MAS, there is also a lack of specific guidelines, and treatment often references medications used for HLH, including traditional agents such as glucocorticoids, etoposide, cyclosporine or tacrolimus, intravenous immunoglobulin (IVIG), plasma exchange (20), etc. or the combination use of biologics such as anakinra (21), IL-18 binging protein (IL-18BP) (22), MAS-825 (23), emapalumab (24), and JAK inhibitors (25). TNF-α monoclonal antibodies or tocilizumab are not recommended (26, 27). Despite the progress made, treating individuals with similar clinical symptoms in AIIRD-MAS requires a comprehensive understanding of the different triggers and pathogenic mechanisms. Early recognition is crucial to avoid prolonged high-inflammatory states leading to multiorgan failure. This review article explores the main cytokines involved, the major pathological cell types, the heterogeneity observed in the preclinical mice model of AIIRD-MAS, and the progress in treatment related to AIIRD-MAS.

## 2 Categorization of HLH syndrome

**Figure 1:**
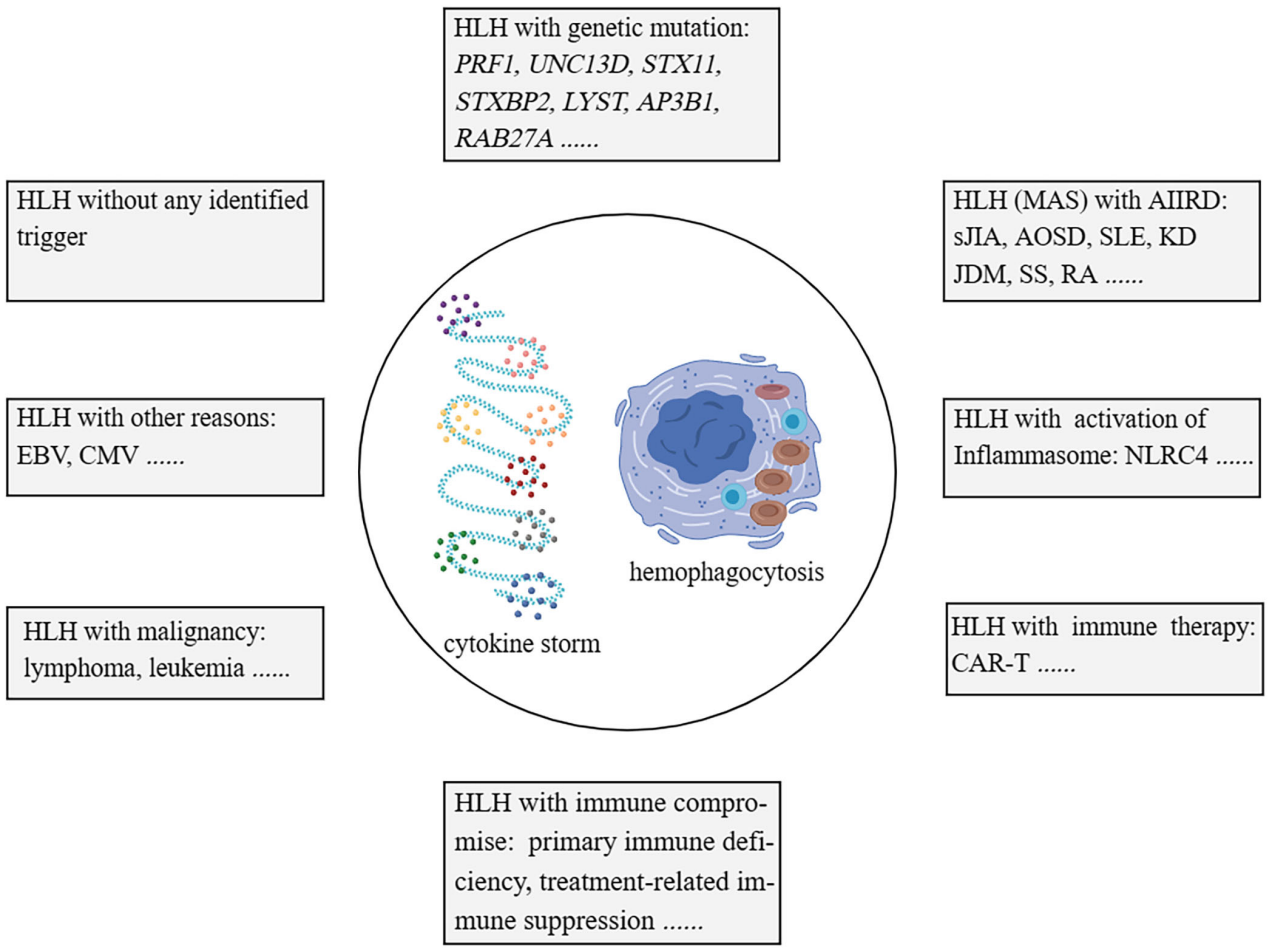
Categorisation of HLH syndrome. HLH with monogenic defects (fHLH); HLH with AIIRD; HLH induced by excessive activation of Inflammasome; HLH with immune impairment; HLH with immune activation therapies; HLH associated with malignancies; HLH induced by infections; HLH without any identified trigger. HLH, hemophagocytic lymphohistiocytosis. fHLH, Familial HLH. AIIRD, autoimmune inflammatory rheumatic disease.

HLH (hemophagocytic lymphohistiocytosis) disease has traditionally been classified into primary and secondary categories. However, as our understanding of the complexity of genetic factors in HLH has grown, solely relying on genetic factors for differentiation has become less suitable. HLH can be categorized into the following groups: 1). Familial HLH (fHLH) with defined monogenic defects (e.g. PRF1 (Perforin 1), UNC13D (Unc-13 homolog D), STX11 (Syntaxin 11), STXBP2 (Syntaxin binding protein 2), LYST (Lysosomal trafficking), AP3B1 (Adaptor-related protein complex 3, beta 1), and RAB27A (Ras-related protein Rab-27A)); 2). AIIRD-MAS (e.g. sJIA, AOSD, SLE, KD, and JDM); 3). HLH induced by excessive activation of the inflammasome (e.g. genetic defects in NLRC4 (NOD-like receptor family, pyrin domain-containing protein 4)); 4). HLH with immune impairment (primary immunodeficiencies or immune suppression-related); 5). HLH occurring after immune activation therapies (e.g. Chimeric Antigen Receptor T-Cell Immunotherapy (CAR-T), cell therapy); 6). HLH associated with malignancies (e.g. lymphoma); 7), HLH induced by infections (e.g. EBV, CMV); 8), HLH without any identified trigger (1, 2, 28–31) (**Figure 1**). This review article primarily focuses on AIIRD-MAS.

## 3 Cytokine storms involved in AIIRD-MAS

**Figure 2:**
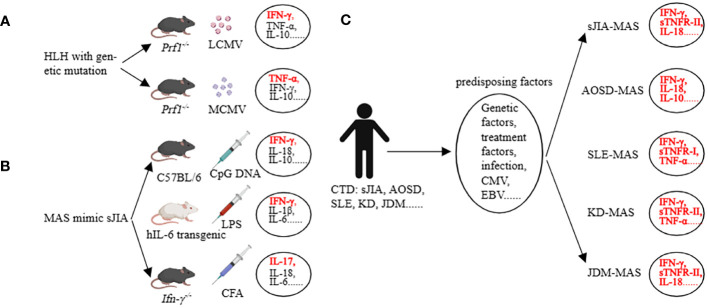
Cytokine storm in preclinical models (HLH, MAS) and in clinical patients (MAS). **(A)** Many cytokines are involved, among which IFN-γ and TNF-a are the main pathogenic factors in the corresponding HLH models. **(B)** Many cytokines are involved, among which IFN-γ and IL-17 are the main pathogenic factors in the corresponding MAS models. **(C)** The main cytokines involved in AIIRD-MAS. HLH, hemophagocytic lymphohistiocytosis. AIIRD, autoimmune inflammatory rheumatic disease. MAS, Macrophage activation syndrome.

Before discussing AIIRD-MAS, we first focus on the main types of cytokines involved in the HLH with genetic defects (32). In the prf1^-/-^ mice infected with lymphocytic choriomeningitis virus (LCMV), all 8 criteria of HLH-2004 can be achieved (14). In this model, IFN-γ is considered the main pathogenic factor, accompanied by elevated levels of TNF-α, IL-6, IFN-α, IL-10, IL-12, IL-33, IL-18, GM-CSF (granulocyte-macrophage colony-stimulating factor), M-CSF, CXCL10 (C-X-C motif chemokine ligand 10), CCL5 (C-C motif chemokine ligand 5), etc. (32–34). However, when prf1^-/-^ mice are infected with murine cytomegalovirus (MCMV), 4 criteria of HLH-2004 can be achieved including splenomegaly, cytopenia, hemophagocytosis, and low natural killer (NK)-cell activity. TNF-α is considered the main pathogenic factor, accompanied by elevated levels of IFN-γ, IL-10, etc. (35, 36) (**Figure 2A**). This indicates that even within the same genetic background, different infection triggers can result in different cytokine profiles and the involvement of different cytokines as main effector molecules (32, 35, 36).

The mice models of sHLH primarily focused on sJIA-MAS, including the repeated CpG injection model (37), hIL-6 transgenic mice combined with Toll-like receptor (TLR) stimulation model (38), IFN-γ knockout mice combined with complete Freund’s adjuvant (CFA) stimulation model (39), IL1RN (Interleukin-1 receptor antagonist) deficiency model (40), Tsc2 (tuberous sclerosis complex 2) knockout mice model (41), NSGS mice with xenograft model (42, 43), and the CD40 intervention model (44). In the repeated CpG injection model, an elevation in IFN-γ, IL-18, IL-6, IL-10, IL-33, and IL-12 can be observed (45). Interestingly, we have assessed the role of IL-33 in this model and found that the IL-33/ST2 axis has little impact in this model, whereas IFN-γ and IL-10 are relatively important (46). The hIL-6 transgenic mice combined with the TLR stimulation model have demonstrated the main role of IFN-γ and IL-6 in the disease, accompanied by elevations in IL-1β, IL-18, TNF, CXCL9, and CXCL10 (38, 47). The IFN-γ knockout mice combined with the CFA stimulation model have shown that the lack of IFN-γ, in conjunction with IL-17, can induce certain phenotypes of the disease (**Figure 2B**). Additionally, G-CSF plays a dual role in both pathogenesis and protection in this model (39, 48). Other models have also implicated various factors such as IL-1, S100A8/A9, MIP, etc. (41, 42, 49, 50). These data demonstrate the heterogeneity of the disease, as different cytokine profiles can lead to similar clinical manifestations.

Some clinical data partially support the findings from mice studies. In a study involving children with different rheumatic diseases complicated by MAS, it was found that excessive production of IFN-γ, IL-18, and TNF-α was closely associated with MAS. Additionally, sTNFR-1 could serve as a diagnostic biomarker for SLE-MAS, IL-18 as a diagnostic biomarker for JDM-MAS, and sTNFR-II as a diagnostic biomarker for KD-MAS and sJIA-MAS (5). Another study showed that AOSD-MAS expressed higher levels of IL-1α, IL-1β, IL-1Ra, IL-2Ra, IL-6, IL-10, IL-17A, IFN-γ, G-CSF, MCP-1, MIP-1α, and SCF compared to AOSD. In addition, IFN-γ, IL-10, ferritin, and systemic score were able to differentiate the occurrence of AOSD-MAS (51). Another study evaluating SLE and SLE-MAS, as well as AOSD and AOSD-MAS, found that the types of cytokines involved were similar. They concluded that the occurrence of MAS is driven by enhanced underlying cytokine abnormalities rather than a MAS-specific cytokine profile. They also emphasized that type I interferon may play a role in the development of MAS in SLE patients (52). Additionally, IL-18 is not only a risk factor for MAS in patients with sJIA, but also a marker of disease activity in MAS-sJIA (53). Even when other markers are normalized, the concentration of IL-18 remains elevated (54) (**Figure 2C**). These studies suggest that the cytokine profiles of MAS vary depending on the underlying diseases. Understanding the key cytokines involved in AIIRD-MAS can be beneficial for disease recognition and treatment.

## 4 The mechanisms underlying AIIRD-MAS

**Figure 3:**
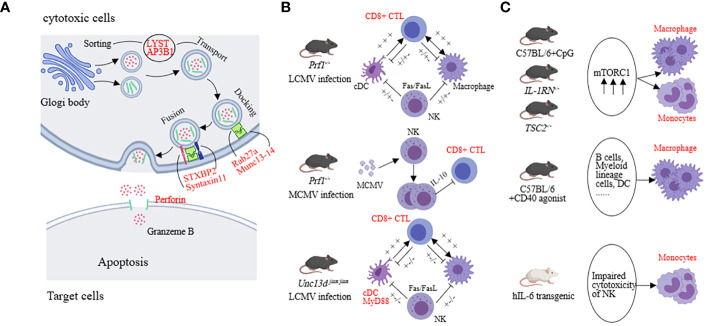
The underlying mechanisms in preclinical models (HLH, MAS). **(A)** The molecular mechanisms mainly involve factors associated with vesicle fusion, including PRF1, UNC13D, STX11, STXBP2, LYST, AP3B1, and RAB27A. **(B)** Both LCMV-infected prf1-/-mice and McMv-infected prf1-/-mice mainly affected CD8+ CTL, but the former released more IFN-γ and the latter was negatively regulated by NK cells. cDC was the predominant pathogenic cell in LCMV-infected Unc13d ^jinx/jinx^ mice. **(C)** Monocytes and macrophages are the main pathogenic cells in the sJIA-MAS mouse model.

We first discuss the relevant mechanisms in HLH with genetic defects which are believed to be associated with defects in the release of cytotoxic granules (involving processes such as vesicle sorting, transport, membrane fusion, and perforation of the target cell membrane) related to PRF1, UNC13D, STX11, STXBP2, LYST, AP3B1, and RAB27A (55–62) (**Figure 3A**). Interestingly, activating mutations in the NLRC4 inflammasome have been found to also lead to recurrent MAS in patients. Although not fully understood, excessive production of IL-18 seems to play a role in this process (63). In addition to the molecular-level mechanisms, the main pathogenic cell subsets have also been extensively studied. In the LCMV-prf1^-/-^ model, CD8+ T cells are the main pathogenic cells. NK cells and CD4+ T cells are considered less important (33, 64). In the MCMV-prf1^-/-^ mode, CD8+ T cells also play an important role, but NK cells proliferate and express IL-10 in response to MCMV stimulation, exerting a negative regulatory function (36). In the LCMV-infected Unc13d ^jinx/jinx^ mice, excessive activation of cDCs plays a crucial role. Blocking IFN-γ and TNF-α cannot halt the disease, and blocking IFN-γ may even increase the mortality rate. However, blocking MyD88 can improve symptoms (65, 66) (**Figure 3B**). Furthermore, in LCMV-infected Stx11^-/-^ mice, CD8+ T cells rather than NK cells play a major pathogenic role. However, residual T cell cytotoxicity can still be detected in this model (67). Interestingly, implantation of only 10 to 20% perforin-expressing T cells is sufficient to reestablish normal cytotoxic function and protect Prf1^-/-^ mice from HLH-like disorders (68).

In the model of sHLH, such as repeated CpG administration, T cells, B cells, NKT cells, and NK cells are not the primary effector cells driving the disease. Impressively, activation of myeloid cells exhibits a pronounced pathogenic advantage. Inhibiting the mammalian target of rapamycin (mTOR) activation in myeloid cells with rapamycin can alleviate the severity of the disease (41, 45, 69, 70). In the hIL-6 transgenic mouse model combined with TLR stimulation, impaired NK cell cytotoxicity is considered a key pathogenic mechanism. For example, upon stimulating the NK cells in the spleen of hIL-6 TG mice with poly(I:C), the expression of perforin and lysozyme B in NK cells was decreased while the degranulation function remained normal (71). In the IFN-γ knockout combined with the CFA intervention model, NK cell number is decreased and the expression of perforin and granzyme B complexes is reduced, leading to impaired cytotoxicity (72). The IL-1RN deficiency model reflects sJIA-like symptoms. Excessive activation of mTORC1 in monocytes/macrophages may contribute to the pathogenesis. Similarly, in mice with TSC2 deficiency, spontaneous activation of mTORC1 can induce MAS-like symptoms (41, 69). In the NSGS mice with xenograft model, MAS-like symptoms are also driven by myeloid cells. NK cells have cytotoxic activity against hyperactivated myeloid cells, providing a protective role in this model (43). CD40-induced MAS-like symptoms are primarily caused by macrophages because the ablation of other types of immune cells does not completely reverse the inflammatory features (44) (**Figure 3C**). These findings suggest that impaired cytotoxic function of NK cells and excessive activation of monocytes or macrophages play a significant role in the sHLH model. This suggests that in disease models with a higher genetic predisposition, CD8+ cytotoxic T cells play a primary role, whereas, in secondary models, impaired NK cell cytotoxic function and excessive activation of myeloid cells have a predominant pathogenic effect.

In studies on human populations, it is interesting to note that recently, a unique pathogenic cell subset, namely the CD4dim CD8+ T cells, which is significantly increased and expresses markers of activation/exhaustion such as CD38, human leukocyte antigen DR (HLA-DR), CD25, programmed cell death protein 1 (PD-1), CD95, and IFNγ, has been found in the peripheral blood of patients with MAS/sHLH (73). It is worth noting that this subset can distinguish activated sJIA patients from MAS/sHLH patients (with a sensitivity of 90% and specificity of 85.2%), and is associated with disease severity. Importantly, the quantity of this subset is not influenced by glucocorticoids, providing an opportunity for early identification of MAS/sHLH patients and better management with early treatment (73). The pathogenesis and features in MAS-AIIRD are very important, but there are still many unknowns at present, which require further studies.

## 5 Characteristics of AIIRD-MAS

AIIRD-MAS is a rare but life-threatening complication. Failure to identify and treat it early with appropriate measures can significantly increase hospitalization time and mortality rate in patients. Therefore, it is important to understand the characteristics of MAS in different rheumatic disease backgrounds. This review article mainly elucidates the characteristics of sJIA-MAS (74), AOSD-MAS (75), SLE-MAS (76), KD-MAS (77), and MAS associated with other rheumatic diseases [such as IIM (idiopathic inflammatory myopathies), RA (rheumatoid arthritis), SS (Sjögren syndrome), etc.] (78–80), in hopes of improving the ability to understand, diagnose, and treat the AIIRD-MAS (**Table 1**).

**Table 1:** Characteristics in different AIIRD-MAS.

Disease	Major cytokines	Incidence	Mortality	Treatment	Biological agents	Refs
sJIA-MAS	IFN-γ, IL-18, TNF-α, sTNFR-II, etc.	10%-25%	8%-23%	Blocking triggers, GC, etoposide, CsA, plasma exchange, leukocyte removal, IVIG, etc.	Emapalumab, anakinra, ruxolitinib, etc.	(5, 81–84)
AOSD-MAS	IFN-γ, IL-18, IL-10, TNF-α, IL-6, etc.	12%-14%	10%-22%	Blocking triggers, GC, etoposide, CsA, plasma exchange, leukocyte removal, IVIG, etc.	Emapalumab, anakinra etc.	(10, 51, 83)
SLE-MAS	TNF-α, IFN-γ, IFN-α, sTNFR-I, etc.	1.5%-4.6%	5%-35%	GC, etoposide, CsA, Tacrolimus, plasma exchange, IVIG, etc.	Anakinra, ruxolitinib, etc.	(5, 9, 52, 85–87)
KD-MAS	IFN-γ, IL-18, TNF-α, sTNFR-II, etc.	1.1-1.9%	13%	Blocking triggers, GC, etoposide, CsA, plasma exchange, IVIG, etc.	NA	(5, 11, 88)
JDM-MAS	sTNFR-II, IFN-γ, IL-18, etc.	Low	16.7%	Blocking triggers, GC, etoposide, CsA, plasma exchange, IVIG, etc.	NA	(5, 12, 74)

AIIRD, autoimmune inflammatory rheumatic diseases; sJIA, systemic juvenile idiopathic arthritis; AOSD, adult-onset Still’s disease; SLE, systemic lupus erythematosus; KD, Kawasaki disease; JDM, juvenile dermatomyositis; MAS, macrophage activation syndrome; GC, glucocorticoids; CsA, cyclosporin A; IVIG, intravenous immunoglobulin; sTNFR-I, soluble tumor necrosis factor receptor I; sTNFR-II, soluble tumor necrosis factor receptor II; IL-18BP, interleukin-18 binding protein; Refs, references.

### 5.1 sJIA-MAS

sJIA is one of the six subtypes of JIA according to the new classification criteria by PRINTO (Pediatric Rheumatology International Trial Organization). It typically begins in childhood and presents with prominent systemic features, including high fever, rash, joint swelling and pain, and inflammation signs (16). sJIA and AOSD represent homogenous diseases with onset at different ages. The high levels of inflammation have prompted researchers to explore genetic evidence. Studies have found that more than one-third of sJIA patients have single allele mutations in genetically associated HLH genes, such as PRF1, UNC13D, STX11, STXBP2, and RAB27A. In sJIA-MAS patients, there is a significantly higher frequency of rare protein-altering variants in UNC13D, STXBP2, and LYST compared to non sJIA-MAS patients (36% vs 14%) (74, 89). The state of high inflammation also contributes to the relatively high incidence of sJIA-MAS, ranging from 10%-25%, with a mortality rate of 8%-23%. Therefore, early recognition of sJIA-MAS is crucial (9, 81, 82). In the 2016 classification criteria for sJIA-MSA, the ferritin level greater than 684 ng/mL, along with any two of the following criteria, is sufficient to diagnose sJIA-MAS: platelet count abnormality ≤181 × 10^9/L, aspartate transaminase (AST) level >48 U/L, triglycerides >156 mg/dL, and fibrinogen abnormality ≤360 mg/dL (16). However, in relapsed or active sJIA patients, ferritin levels can also exceed 684 ng/mL, making it difficult to differentiate sJIA-MAS. It is worth noting that IL-18 is significantly elevated in sJIA-MAS, with levels >47,750 pg/mL significantly increasing the likelihood of sJIA developing MAS. Another marker, neopterin, is also significantly elevated in sJIA-MAS. Therefore, the combination of IL-18 and neopterin might effectively differentiate sJIA-MAS from relapsed or active sJIA (74, 90, 91). Moreover, patients with sJIA-MAS often have higher levels of IFN-γ and CXCL9 compared to those with active sJIA disease in serum (92). A recent study also identified soluble tumor necrosis factor receptor-II (sTNFR-II) as a diagnostic biomarker for sJIA-MAS (5). Additionally, IL-18 and IFN-γ induce the production of adenosine deaminase 2 (ADA2) in peripheral blood mononuclear cells, and the activity of plasma ADA2 can aid in the rapid diagnosis of MAS in sJIA (93). In terms of treatment, high-dose corticosteroid pulse therapy, along with the induction of remission using drugs such as etoposide and cyclosporine, can be employed. For refractory cases, options include plasma exchange or leukocyte removal. Additionally, biological drugs such as emapalumab, anakinra, and JAK inhibitors can be used to treat difficult cases (83, 84). These data emphasize the importance of effective diagnostic biomarkers for early recognition of the disease and the need for further research to evaluate the safety and efficacy of biological therapies for sJIA-MAS.

### 5.2 AOSD-MAS

AOSD is a rare, multi-genetic, inflammatory autoimmune disease. It is characterized by symptoms such as high fever, rash, and joint pain, and is often accompanied by elevated neutrophil count, hepatosplenomegaly, and lymphadenopathy (94). The incidence of AOSD-MAS is also relatively high, accounting for approximately 12%-14% of AOSD patients, and the mortality rate is high, ranging from 10%-22% (10). Therefore, early recognition of MAS in AOSD patients is crucial in reducing the risk of mortality. Ferritin levels are significantly increased in both AOSD-MAS and active AOSD patients. Prior studies have reported that approximately 31.5% of AOSD patients have serum ferritin levels greater than 10,000 μg/L, while in AOSD-MAS patients, this proportion is 33.3%. However, a study on Still’s disease (SD) suggested that using a serum ferritin threshold of 3,500 μg/L achieved 85% sensitivity and 97% negative predictive value for identifying patients with/without MAS, which indicated that If ferritin was less than 3500 μg/L, MAS was ruled out with high probability. It is worth noting that this study included children and adult patients, so it is not specific for adult patients with Stills disease (75, 95). IL-18 levels are significantly increased in both AOSD-MAS and active AOSD patients, although the levels are higher in AOSD-MAS. However, no significant difference has been found in IL-18 levels between the two groups (75). The occurrence of jaundice is significantly higher in AOSD-MAS patients (33.3%) compared to AOSD patients (2.9%), and its presence is considered to be associated with MAS (75). Splenomegaly and pericarditis are also considered predictors of MAS occurrence (96). Measurement of lymph node metabolic lesion volume using PET/CT can predict the occurrence of MAS (MLV_total_ of LN >62.2, with a sensitivity of 80.0% and specificity of 93.9%) (97). In another study, the sJIA-MAS diagnosis was revised to meet the diagnosis of AOSD-MAS. The revised criteria were ferritin >2810 mg/mL along with any two of the following: platelet (PLT) ≤ 137 × 10 ^9/L, AST > 95 U/L, and fibrinogen ≤ 365 mg/dL. The modified criteria had a sensitivity of 100%, specificity of 93%, positive predictive value of 80%, and negative predictive value of 100% (98). Data on the use of neopterin as a diagnostic marker in AOSD-MAS patients is lacking. Treatment is similar to that of sJIA-MAS. As MAS is an independent risk factor for mortality in AOSD (75), therefore, early recognition and timely treatment of the disease should be prioritized.

### 5.3 SLE-MAS

SLE is an autoimmune disease that affects multiple systems, including the skin, serosal surfaces, joints, kidneys, and central nervous system (99). The incidence of SLE-MAS is relatively low, accounting for approximately 1.5%-4.6% (100). The mortality rate of SLE-MAS ranges from 5%-35% (9). The pathogenesis of SLE-MAS is still unclear, but it is believed to be associated with dysregulation of macrophage-lymphocyte interactions. Additionally, the high expression of TNF-α is considered a characteristic feature of SLE-MAS, and sTNFR-I is high and might be a useful diagnostic marker for SLE-MAS, but further work is needed to clarify its role as a diagnostic tool. These are significantly different from sJIA and AOSD (5, 101). CXCL9 levels are also elevated in SLE-MAS (102). IgM anti-lymphocyte antibody (ALAB) and MEFV gene mutations may also play a role in the SLE-MAS (101). Serum levels of sCD163 positively correlate with SLE-MAS activity, but the possibility of corticosteroid-induced upregulation of CD163 expression cannot be excluded (103). Hydroxychloroquine has been suggested to reduce the risk of MAS in SLE patients (104), and it is speculated that hydroxychloroquine may reduce the production of IL-1, IL-6, and TNF-α, and inhibit TLRs, thereby reducing the likelihood of developing SLE-MAS. High-dose corticosteroids lead to remission in two-thirds of cases (85). For uncontrolled/severe forms of SLE-MAS, consideration should be given to the use of cyclophosphamide or etoposide. For the maintenance of remission, a combination of tacrolimus and corticosteroids is recommended (105). There are also reports on the use of rituximab and anakinra in the treatment of SLE-MAS (86, 87). It is worth noting that the high inflammation associated with SLE-MAS may be related to TNF-α and IFN-γ. However, currently, there is a lack of data on the efficacy of TNF inhibitors (TNFi) in this disease. Considering TNFi may carry a risk of inducing MAS, so caution should be exercised when using them. For emapalumab, the ongoing trial (NCT05001737) is expected to end in September 2025 and the data are currently unavailable. We look forward to more related research in the future.

### 5.4 KD-MAS

KD is a disease characterized primarily by systemic vasculitis, with fever, rash, and lymphadenopathy as the main symptoms. In a small proportion of patients, coronary artery lesions can occur (106). The incidence of KD-MAS is relatively low, accounting for only 1.1-1.9% of KD patients (88), but the mortality rate is high, around 13% (11). MAS can occur at any stage of KD and has a significantly increased occurrence rate in children over 5 years of age (8). It is worth noting that the levels of sTNFR-II and neopterin in the peripheral blood of KD-MAS patients are significantly increased, suggesting the role of TNF-α and IFN-γ in the disease. Another study found that IL-18 can serve as a diagnostic marker for KD-MAS (5). In addition, 69% of KD-MAS patients experience splenomegaly (which is rare in KD patients) (11), and 46% of KD-MAS patients have coronary artery abnormalities. Furthermore, KD-MAS often manifests as resistance to IVIG treatment (107). A study analyzed early predictive factors for KD-MAS occurrence and found that KD patients with a platelet count <110 × 109/L and serum ferritin >548.4 ng/mL were more likely to develop KD-MAS (108). However, due to the currently low diagnostic efficiency, MAS may be underestimated. For the treatment of KD-MAS, in addition to IVIG, corticosteroids and immunosuppressive agents can be added, and refractory cases can be considered for plasma exchange. There is currently a lack of data on the efficacy of biological drugs such as emapalumab and TNFi (which should be used cautiously) in this disease. It is hoped that more research will promote a better understanding of KD-MAS, including its diagnosis and treatment.

### 5.5 Other AIIRD combined with MAS

MAS has also been reported in other types of AIIRD, such as idiopathic inflammatory myopathies (IIMs) (109), RA (110), and SS (80). MAS in IIMs mainly occurs in JDM and dermatomyositis with MDA5 positivity (111), and there are also a few reports of adult dermatomyositis and necrotizing myopathy (112). In this article, we mainly discuss JDM-MAS. There is limited research on JDM-MAS, and a systematic review found that JDM-MAS usually occurs before the final diagnosis of JDM and its incidence may be underestimated (78). Studies on peripheral blood cytokines have found increased levels of sTNFR-II, neopterin, and IL-18 in JDM-MAS patients (5, 74), and IL-18 may be used as a diagnostic marker (5). JDM-MAS patients are relatively difficult to treat and may require combination therapy with high-dose steroids, etoposide, IVIG, cyclophosphamide, or anakinra (109). There are very few reports on RA-MAS. In one case of a fatal RA-MAS patient, high levels of IL-18 and G-CSF were detected in the peripheral blood (113). Another study found that the triggering factor for RA-MAS is lymphoproliferative disorders associated with MTX, and improvement was observed after discontinuing MTX and treatment with high-dose steroids (110). Additionally, it should be noted that TNFi may be a potential trigger for MAS. Considering that RA patients often use TNFi, this factor should be taken into consideration when assessing the condition (79). SS-MAS can also be found in some case reports, but they are very rare and are often caused by infections (e.g. EBV, CMV) or inappropriate use or discontinuation of medications (e.g. zonisamide, corticosteroids, hydroxychloroquine, methotrexate). Patients often show improvement after combination therapy with antiviral agents, IVIG, and steroids. Currently, there is a lack of large-scale data on these diseases. Furthermore, there are a few reports of MAS occurring in conditions such as systemic sclerosis (8, 114) and granulomatosis with polyangiitis (115), but this review article does not elaborate on them in detail.

## 6 Progressions in treatment

With the advent of the era of biological therapy, patients with AIIRD have more treatment options. However, we must also recognize the complexity of the interactions between various cells and cytokines in the aberrant immune system. This may partially explain why treatment with a single biological drug is only effective in some patients or initially effective but then leads to diminished efficacy and drug resistance. Currently, the treatment of patients with AIIRD-MAS is mainly based on clinical experience or reference to treatment methods used in other types of HLH. High-dose methylprednisolone (10–30mg/kg/day maximum 1g/day for 1-3 days then followed by 1mg/kg/day intravenously or orally) is commonly used, and if ineffective, additional medications such as cyclosporine (2-7mg/kg/day), cyclophosphamide, etoposide (50-150mg/m2/dose 1-2 dose/week), intravenous immunoglobulin (IVIG) (0.4–1 g/kg/day for 2–5 days) and plasma exchange have been reported, but there is a lack of prospective studies in this area (20, 116). In addition, it is important to remain vigilant for potential viral infections during the treatment process. Viruses such as EBV, CMV, and other herpes viruses can not only induce virus-associated HLH but also serve as triggering factors in AIIRD- MAS. Therefore, routine screening is necessary. There are effective treatment options for CMV and some herpes viruses, but no effective treatment options are available for EBV. For the treatment of EBV infection in B cells, rituximab can be used, while for EBV infection in T cells or NK cells, a combination of cytotoxic agents is needed. Given the elevation of TNF-α and IL-6 in the disease, therapeutic antibodies targeting these cytokines have been used. The therapeutic efficacy of TNFi in AIIRD-MAS is not clear, which may be due to the simultaneous blockade of cytotoxic signals mediated by TNFR1, pro-inflammatory processes mediated by both TNFR1 and TNFR2, and anti-inflammatory and tissue-protective activities mediated by TNFR2. Therefore, the treatment effect of inhibiting both TNFR1 and TNFR2 depends on the overall net effect (117). On the other hand, blocking TNF also leads to increased release of type 1 interferons and an increased risk of infection (118). The efficacy of anti-IL-6R is also unclear, and some studies have suggested the occurrence of MAS is related to the use of tocilizumab. Furthermore, blockade of IL-6R often masks inflammatory markers and fever, which negatively impacts their use in AIIRD-MAS (27). Other treatment strategies include stimulating inhibitory receptors to promote CD8+ T cell exhaustion and reduce cytokine secretion, clearing antigen-presenting cells such as dendritic cells to block positive feedback loops, blocking the CD40 signaling pathway in macrophages or blocking downstream MyD-88 in myeloid-derived cells, as well as targeting mTOR with rapamycin (69). This review article focuses on the efficacy of emapalumab (6 mg/kg on day 0, followed by 3 mg/kg every 3 days until day 15 and twice weekly until day 28), anakinra (intravenous or subcutaneous 5–10mg/kg/day), ruxolitinib (25 mg/m2/dose, two times per day), IL-18BP (subcutaneous injections of either 80-160mg three times per week) and MAS-825 in AIIRD-MAS (**Table 2**).

**Table 2:** Progressions in treatment of AIIRD-MAS.

Drug	Targeted molecules	Treatment for AIIRD-MAS	Advantages	Side-effects	Refs
emapalumab	IFN-γ	sJIA-MAS, AOSD-MAS	low toxicity, and not experience bone marrow suppression	viral infections, especially cytomegalovirus	(24, 119)
anakinra	IL-1R	sJIA-MAS, AOSD-MAS SLE-MAS	low bone marrow suppression, hepato-toxicity, a short half-life, and not mask infection markers	potential infections, neutropenia	(21, 120, 121)
ruxolitinib	JAK1, JAK2	sJIA-MAS, SLE-MAS	oral formulations with short half-lives.	Jak2 potential toxicity, narrow therapeutic window	(25, 84, 86, 122)
IL18-BP	IL-18	NA	NA	ISRs, upper airway infections, arthralgia	(123)
MAS-825	IL-1β, IL-18	NA	NA	NA	(23)

sJIA, systemic juvenile idiopathic arthritis; MAS, Macrophage activation syndrome; AOSD, adult-onset Still’s disease; SLE, systemic lupus erythematosus; JAK1, Janus Kinase 1; JAK2, Janus Kinase 2; IL-18BP, interleukin-18 binding protein; Refs, references; ISRs, injection site reaction; NA, not available.

### 6.1 Anti-IFN-γ monoclonal antibody (emapalumab)

Numerous studies have revealed a significant role of IFN-γ in HLH or MAS, and neutralizing antibodies against IFN-γ have shown therapeutic effects in preclinical models (31, 69). However, there are also a few preclinical models where blocking IFN-γ has worsened the condition or IFN-γ knockout induces MAS-like symptoms (66). Histopathological studies in MAS have shown a large number of IFN-γ-producing T cells close to activated phagocytic tissue macrophages (124). Additionally, significantly elevated levels of neopterin, CXCL9, and CXCL10 can be detected in the peripheral blood of MAS patients (124). A groundbreaking study evaluated the therapeutic effect of emapalumab in primary HLH. After 2 months of treatment, the response rate was 63% in patients who had previously received other drug treatments, and 65% in patients receiving initial treatment, which was higher than the response rates in the HLH-94 protocol (59%) and the HLH-2004 protocol (51%). In contrast, patients treated with emapalumab had low toxicity and did not experience bone marrow suppression although potential infections should be noted (119). This study prompted research on the use of emapalumab for the treatment of MAS. It was found that patients with sJIA-MAS or AOSD-MAS showed rapid improvement after treatment with emapalumab, with a median time to resolution of 25 days, and a significant acceleration of corticosteroid tapering. During the treatment, attention should be paid to viral infections, especially cytomegalovirus, and regular viral screening should be performed (24). In addition, it is noteworthy that a clinical trial (NCT05001737) assessing the safety, tolerability, and efficacy of emapalumab in SLE-MAS is currently ongoing. These data suggest the potential of emapalumab in the treatment of MAS-sJIA or MAS-AOSD and further evaluation of its role in other AIIRD- MAS is warranted.

### 6.2 Antagonist of IL-1R (anakinra)

IL-1β is found to be elevated in some preclinical and clinical studies, but due to technical difficulties and variability, there is often a lack of comparability between different laboratories (21). Furthermore, serum IL-1β is not a reliable marker of disease activity, as serum IL-1β levels in active sHLH patients are within normal range and are not associated with treatment outcomes, although the possibility of increased IL-1β in the local microenvironment cannot be ruled out (21). These findings suggest that anakinra may not necessarily be effective in MAS. However, early studies have shown that subcutaneous anakinra has been efficacious in treating patients with MAS (125–128). Subsequent research has further revealed that intravenous administration of Anakinra is more advantageous in treatment when patients present with subcutaneous edema, severe thrombocytopenia, or neurological impairment (21). Anakinra has been confirmed to be effective in sJIA-MAS and has improved the survival rates of other AIIRD-MAS, including SLE-MAS (120, 121). In a retrospective study assessing the therapeutic outcomes in 44 pediatric patients with sHLH, it was found that the use of anakinra within 5 days of hospitalization was associated with a lower mortality rate (128). The use of anakinra in children with AIIRD-MAS is associated with better clinical outcomes, with an overall survival rate of 73%, higher than the survival rate of HLH patients treated with etoposide (56%) in which sHLH patients accounted for 78% (21, 128). These data suggest that anakinra can effectively alleviate the symptoms of MAS and reduce mortality. Anakinra has low bone marrow suppression and hepatotoxicity, a short half-life, and does not mask infection markers, making it a good choice for the treatment of AIIRD-MAS, however, monitoring for potential infections and avoiding neutropenia is necessary.

### 6.3 JAKi (ruxolitinib)

JAK inhibitors are small-molecule drugs. Ruxolitinib, which targets JAK1 and JAK2 kinases, is primarily used for the treatment of myelofibrosis and has also been approved for the treatment of patients with acute and chronic graft-versus-host disease (129). Many studies found that ruxolitinib has significant advantages in treating prf1^-/-^ mice infected with LCMV and can reverse the high inflammatory state (130–134). However, another study has also indicated that ruxolitinib has a relatively narrow therapeutic window in this model, and inhibition of JAK2 may have potential toxicity (122). To further validate the efficacy of ruxolitinib, a clinical study on adult secondary HLH found that among 5 enrolled patients, 3 achieved a complete response and 2 achieved a partial response. The use of ruxolitinib reduced the dose of corticosteroids, and all patients survived after 2 months. However, there was 1 case of relapse and 1 case of drug intolerance (neuropathic foot pain) (25). Another retrospective study found that combining ruxolitinib with dexamethasone showed better efficacy in newly diagnosed secondary HLH in adult patients (overall response rate 87.5%, complete response 50%, partial response 37.5%, 2-month overall survival 75%, 6-month overall survival 50%) (130). Some case reports or case series have also documented studies showing that other types of JAK inhibitors (e.g. baricitinib, tofacitinib, and peficitinib) have been effective in treating some refractory Still’s disease patients (135–137). These patients have a higher risk of developing MAS. The use of JAK inhibitors may be helpful for some patients who are dependent on high doses of corticosteroids and may have a corticosteroid-sparing effect. Overall, JAK inhibitors have potential advantages in treating AIIRD-MAS, which is characterized by cytokine storms, as JAK is downstream of multiple cytokine signaling pathways. Additionally, JAK inhibitors are available in oral formulations with short half-lives. However, further research and data are still needed to assess the timing and conditions for the rational use of JAK inhibitors in this disease. Furthermore, considering the potential toxicity of JAK2 in mouse models, the specific role of JAK1 in AIIRD-MAS needs to be evaluated.

### 6.4 IL-18BP

IL-18 is a member of the IL-1 family and can induce IFN-γ production. It is significantly elevated in patients with sJIA, AOSD, MAS, and autoimmune inflammatory diseases (22). Unlike IL-1β, IL-18 can be detected in peripheral blood, and the majority of IL-18 binds to IL-18BP. Therefore, the total IL-18 is generally measured, and extremely high total IL-18 levels are associated with MAS susceptibility (53, 138). However, for sJIA and AOSD patients with only joint involvement, total IL-18 is lower (139). In addition, total IL-18 is also lower in fHLH patients and viral sepsis, but with extremely high levels of CXCL9 (140). The IL-18 levels in MAS patients are usually several orders of magnitude higher than the most severe fHLH patients, and the levels of IL-18BP in fHLH or other forms of HLH patients are generally higher than those in MAS patients. Similar to CXCL9, the levels of IL-18BP are associated with IFN-γ response (140). Although IL-18 has multiple sources, monocytes or macrophages appear to be the most likely source of IL-18 overproduction in MAS (22, 141). Although the specific mechanism of IL-18 in MAS is not clear, it at least partially involves excessive production of IFN-γ and excessive activation of CD8+ T cells (142, 143). Animal experiments have revealed a protective role of IL-18BP in a repetitive TLR-9 model (144, 145), but this drug is not currently available in routine clinical practice, and currently a lack of clinical studies on the therapeutic effect of IL-18BP in MAS, although anti-inflammatory treatment with IL-18BP in AOSD was shown to be effective in a phase II clinical trial (123). Further research is needed to clarify the mechanisms of IL-18 in MAS and to develop more relevant therapeutic targets.

### 6.5 MAS-825

MAS-825 is a dual-specific monoclonal antibody targeting IL-1β and IL-18. For MAS, IL-18 plays a significant role in its pathogenesis, while IL-1β may also be actively involved. Their production is closely related to the activation of inflammasomes. Detailed information can be found in the above discussion on the roles of anakinra and IL-18BP in treatment. Therefore, theoretically, the use of dual-specific monoclonal antibodies for treatment is effective, but currently lacking relevant clinical studies. A recent study described the treatment outcome of MAS-825 in a patient with sJIA-LD (sJIA associated with interstitial lung disease), which is a highly morbid subset of sJIA (23). The results showed significant improvement in the patient’s pulmonary symptoms and lung inflammation. After 10 months of treatment, the patient was able to completely discontinue systemic corticosteroids and other biologics, with no reported drug side effects. MAS-825 is a novel treatment approach that needs further evaluation for its therapeutic effects in AIIRD-MAS.

## 7 Discussion

MAS is a rare but serious complication of AIIRD that often leads to patient death. Early recognition and appropriate treatment are closely related to saving patient lives and reducing hospital stays. This article summarizes the current knowledge on cytokine storms, potential mechanisms, and treatment advances related to AIIRD-MAS. Compared to classical genetic defects in HLH, the pathogenesis of MAS may be related to multiple factors such as inappropriate treatment, viral infections, and genetic factors. These factors lead to increased production of IFN-γ or enhanced response to IFN-γ by various immune cells, as well as the production of various pro-inflammatory factors. This results in impaired cytotoxic function of CD8+ CTLs and NK cells, clearance disorders of antigen-presenting cells, and excessive activation of myeloid cells of monocytic origin (including activation of mTORC1 and CD40L-CD40 signaling pathways). Ultimately, multi-organ involvement (especially in the hematopoietic system, liver-spleen-lymph node system, and nervous system) occurs. In terms of treatment, in addition to the traditional combination of glucocorticoids with etoposide, cyclosporine, and plasma exchange, with the advent of biologics, some new treatment methods have gradually emerged, such as IFN-γ monoclonal antibodies, IL-1R antagonists (anakinra), JAK inhibitors (ruxolitinib), and IL-18R binding proteins. However, we also need to understand the common pathways of the disease and the heterogeneity in different disease backgrounds to provide a scientific basis for better treatment. On the other hand, the importance of clinical research should also be considered, especially for difficult-to-treat MAS, induction and maintenance strategies for remission, steroid tapering, and treatment after relapse. More research is expected to provide direction for the treatment of AIIRD-MAS.

## 8 Conclusion

With the continuous understanding of AIIRD-MAS and the advent of the biological drugs era, we have gained a deeper understanding of the disease and made more breakthroughs in treatment options. However, it is still important to note the commonalities within the same category of diseases as well as the heterogeneity among different patients and disease backgrounds. Only by grasping these factors can we effectively improve treatment outcomes. Therefore, further research is needed in the future to provide more guidance for the treatment of AIIRD-MAS.

## Author contributions

YD: Writing – original draft, Writing – review & editing. TW: Writing – review & editing. HW: Supervision, Writing – review & editing.
